# *Scutellaria baicalensis* straw improves nutritional and flavor quality of mutton via regulating the expression of IMP-related genes

**DOI:** 10.3389/fvets.2026.1928789

**Published:** 2026-09-23

**Authors:** Yuxia Yang, Jiaojiao Gao, Minghui Zhang, Liangzhong Hou, Tongjun Guo

**Affiliations:** 1 Institute of Feed Research, Xinjiang Academy of Animal Science, Urumqi, China; 2 Xinjiang Key Laboratory of Herbivorous Livestock Feed Biotechnology, Xinjiang Academy of Animal Science, Urumqi, China

**Keywords:** amino acids, bainuburke rams, fatty acids, inosine monophosphate-related genes, meat quality, *Scutellaria baicalensis* straw

## Abstract

*Scutellaria baicalensis* straw (SBS) is a potential feed ingredient, but its optimal inclusion level for improving mutton quality remains unclear. This study examined the influence of dietary SBS supplementation on meat quality, fatty acid profile, amino acid composition, inosine monophosphate (IMP) content, and the expression of IMP-related genes in the longissimus muscle of rams. Sixty Bainuburke lambs (4–5 months old, initial body weight 27.08 ± 3.12 kg) were randomly allocated to six groups (*n* = 10 per group) and fed isonitrogenous and isoenergetic pelleted diets supplemented with 0, 6, 12, 18, 24%, or 30% SBS on a dry matter basis. The feeding trial lasted 80 days, including a 10-day adaptation period followed by a 70-day experimental period. The 24% SBS group (SBS24) showed significantly higher muscle pH and redness (a^*^ value), and lower cooking loss compared to the control (CON, 0% SBS; *P* < 0.05). SBS24 also increased crude protein content, total essential and non-essential amino acids, and IMP content (*P* < 0.05). For intramuscular fatty acid composition, the SBS24 group achieved remarkable reductions in saturated fatty acids (SFA), primarily palmitic acid (C16:0) and stearic acid (C18:0), while dramatically raising the concentrations of health-beneficial unsaturated fatty acids including oleic acid (C18:1n9c), linoleic acid (C18:2n6c) and α-linolenic acid (C18:3n3) (*P* < 0.01). Furthermore, SBS24 up-regulated *PRPS1, ADSL, ATIC*, and *AMPD1* (IMP-related genes) while down-regulating *ADSSL1* and *IMPDH1* (*P* < 0.05). Dietary inclusion of 24% SBS improves mutton nutritional quality by enhancing protein deposition, beneficial fatty acids, flavor-related amino acids, and IMP metabolism. Therefore, 24% SBS is recommended for Bainuburke rams production.

## 1 Introduction

With continuous upgrades to residents' living standards, consumers place greater emphasis on the freshness, nutritional value, and taste of their food. Flavor serves as a crucial indicator of meat freshness, and inosine 5′-monophosphate (IMP) is closely associated with the umami perception in livestock and poultry meat (1). Studies have investigated the mass fraction of IMP and its influencing factors in the muscles of animals such as chickens (2), swine (3), and fish (4, 5). These studies not only reveal differences in IMP accumulation among species but also elucidate the effects of breed, management practices, and post-slaughter handling on its content. In animal organisms, purine metabolism pathways dominate IMP generation and degradation processes, covering *de novo* synthetic routes and salvage pathways, while IMP breakdown produces adenosine monophosphate (AMP) and guanosine monophosphate (GMP) (6), primarily involving ^*^denovo^*^ and salvage synthesis pathways, while catabolism entails degradation into AMP or GMP. Relevant research (7, 8) demonstrates that IMP content is significantly and positively correlated with the expression levels of PRPS1, ADSL, ATIC, and AMPD1 genes, yet significantly and negatively correlated with the expression of ADSSL1 and IMPDH1 genes. The expression levels of these genes regulate IMP content in meat, thereby indirectly influencing tenderness, juiciness, flavor, and freshness. However, existing research has predominantly focused on poultry, monogastric animals, and aquatic species, with relatively limited studies on the metabolic regulation of IMP in ruminant muscle. Given that mutton is a significant component of meat consumption in China and is highly valued for its unique flavor and nutritional profile, this knowledge gap warrants further investigation.

*Scutellaria baicalensis* (also known as Chinese skullcap) is a perennial herbaceous plant of the Lamiaceae family (9). Its dried root is a traditional and widely used raw material in traditional Chinese medicine, renowned for its heat-clearing, detoxifying, antibacterial, and anti-inflammatory effects (10, 11). During the cultivation and processing of *S. baicalensis*, the harvesting of roots generates a massive amount of aboveground stems and leaves, known as *Scutellaria baicalensis* straw (SBS). The annual production of *S. baicalensis* in China is enormous, with the straw yield alone reaching approximately 63,000 tons per year (12). These straws are typically discarded or burned as agricultural waste, which not only results in resource waste but also causes severe environmental pollution. However, recent studies have shown that SBS is not a valueless “waste.” From a nutritional perspective, the crude protein content of SBS ranges from 6.08% to 7.36%, which is higher than that of corn straw and wheat straw; meanwhile, it is rich in cellulose, minerals, and vitamins (13). Its chemical composition is highly similar to that of the root, being rich in flavonoids such as baicalein, baicalin, and wogonin (14, 15), demonstrating immense potential as a high-quality feed resource. In terms of application in ruminants, Ding et al. (16) indicated that dietary baicalin supplementation can significantly increase the average daily gain of fattening Hu sheep, and significantly improve rumen fermentation parameters and overall health status through the dual modulation of immune function and the gastrointestinal microbiome-metabolic crosstalk. Furthermore, another study confirmed that feeding a 1% aqueous extract of *S. baicalensis* root not only promotes the growth of rabbit fish (*Siganus fuscescen*s) but also effectively improves its muscle amino acid and fatty acid profiles (e.g., increasing the levels of beneficial fatty acids such as docosahexaenoic acid and decreasing triglyceride content) (17).

In recent years, significant progress has been made in research on IMP metabolism and flavor regulation in sheep. In terms of dietary regulation, Wang et al. (18) found that oat forage supplementation increased the levels of IMP and flavor-related amino acids in the muscles of Small-tail Han sheep. Through multi-omics analysis, Zhan et al. (19) demonstrated that fermented Lycium barbarum residue significantly increased the IMP content in the longissimus dorsi muscle of fattening Tan sheep and identified multiple differentially expressed genes regulating lipid and amino acid metabolism, such as FOXO1 and COL3A1. At the molecular mechanism level, Jin et al. (20) revealed that signaling pathways such as AMPK regulate the metabolism of flavor compounds in Tibetan sheep; Song et al. (21) elucidated a multi-gene synergistic network mediating mutton quality traits through age and sex. Additionally, species comparisons have revealed that the IMP content in Tibetan sheep is significantly higher than that in yellow cattle and goats (*P* < 0.05). However, current research on ruminants still exhibits distinct limitations. First, most studies remain at the descriptive level of phenotypic determination and differentially expressed gene screening, lacking systematic reports on the coordinated expression patterns of key IMP metabolic genes (e.g., PRPS1, ADSL) in sheep muscle and their quantitative relationship with IMP deposition. Second, interventional studies utilizing specific functional agricultural by-products to actively regulate the IMP metabolic pathway in sheep remain extremely scarce. Furthermore, there is a significant disparity in the research depth of IMP metabolism between sheep and goats: sheep research has advanced to multi-omics-level gene screening and pathway analysis, whereas goat research remains largely confined to phenotypic determination and association analyses of a few major genes like ADSL, lagging significantly behind sheep in research depth. Therefore, this study focuses on sheep to fill the knowledge gap regarding the active regulation of IMP metabolism in ruminants.

Based on these research gaps, this study establishes the regulation of the IMP metabolic pathway in the longissimus dorsi muscle of Bayinbuluke rams by SBS as the core innovative focus. Using SBS as a functional roughage, six dietary gradient levels (0, 6, 12, 18, 24, 30%) were set for Bayinbuluke rams to systematically investigate the regulatory effects of SBS on the expression profiles of key IMP metabolic genes in the longissimus dorsi muscle, and to perform correlation analyses with IMP content and meat quality indicators. The hypothesis of this study is that SBS will significantly affect mutton quality and the expression of IMP-related genes, and that an optimal supplementation ratio exists. The objective is to determine the optimal SBS supplementation level for improving meat quality. This study not only provides a theoretical basis for the industrial application of SBS in high-quality mutton production but also holds great significance for promoting the resource utilization of agricultural and pastoral by-products and the development of sustainable animal husbandry.

## 2 Materials and methods

This investigation was conducted at the Alimu Breeding Cooperative (Heshuo, Xinjiang, China) (latitude 42.30°N, longitude 87.14°E) under the following environmental conditions: altitude 1794 m, mean annual rainfall 98.7 mm, and mean temperature 8.6 °C. All animal raising, slaughter and sampling procedures strictly complied with the Chinese Laboratory Animal Care and Use Guidelines, and the whole experimental scheme obtained ethical approval from the Animal Care Committee of Xinjiang Academy of Animal Science (Approval No. 5 20231228).

### 2.1 Experimental materials

The SBS used in this experiment consisted of naturally air-dried stems and leaves harvested after *Scutellaria baicalensis* root collection in autumn 2022. The raw material was sourced from Alimu Breeding Cooperative, consistent with the raw material described in our previously published trial (12). Complete nutritional indicators of SBS are displayed in Table 1.

**Table 1:** Nutritional composition of SBS (DM basis %).

Items	Nutritional content
Dry matter (DM)	96.54
Crude protein (CP)	6.30
Ether extract (EE)	2.12
Neutral detergent fiber (NDF)	60.82
Acid detergent fiber (ADF)	42.54
Crude ash (Ash)	7.88
Calcium (Ca)	0.79
Phosphorus (P)	0.17

### 2.2 Experimental design, test diets, and management

The experimental design and animal management protocols were identical to those described in our earlier study (12). Briefly, sixty 4–5-month-old Bainuburke rams with an initial live weight of 27.08 ± 3.12 kg were randomly allocated into six independent groups, each containing 10 experimental animals. Every group received fully mixed pelleted rations with gradient SBS additions (0, 6, 12, 18, 24, 30% DM basis). All diets were formulated to maintain equal crude protein and metabolizable energy levels following the industry standard NY/T816-2021. The feeding period lasted 80 days, including a 10-day adaptation period. Detailed diet composition, feeding management, and housing conditions were identical to those previously reported. The experimental diets were analyzed for chemical composition and nutrient content using standard laboratory methods and verified against established feed composition tables (Table 2).

**Table 2:** Composition and nutrient levels of the diet (DM basis, %).

Items	Control	SBS6	SBS12	SBS18	SBS24	SBS30
Ingredients						
Alfalfa hay	14.73	13.42	12.34	10.88	9.47	7.82
Wheat stalk	25.28	20.57	15.66	11.11	6.54	2.18
SBS	0.00	6.00	12.00	18.00	24.00	30.00
Corn	33.74	33.73	33.83	33.74	33.73	33.72
Wheat bran	7.18	7.19	7.17	7.23	7.18	7.03
Cottonseed meal	14.96	14.96	14.88	14.92	14.97	15.14
NaCl	1.00	1.00	1.00	1.00	1.00	1.00
NaHCO_3_	1.61	1.61	1.61	1.61	1.61	1.61
Premix	1.50	1.50	1.50	1.50	1.50	1.50
Total	100.00	100.00	100.00	100.00	100.00	100.00
Nutritional level						
ME/(MJ/kg)	9.20	9.30	9.20	9.22	9.22	9.16
CP	13.34	13.42	13.34	13.38	13.36	13.34
EE	2.36	2.42	2.48	2.54	2.60	2.65
NDF	44.82	43.88	44.76	44.60	44.59	45.17
ADF	27.49	27.45	27.30	26.24	27.16	28.53
Ca	1.27	1.38	1.49	1.53	1.51	1.59
P	0.38	0.42	0.38	0.36	0.39	0.40

Per kilogram of commercial premix contains the following nutrient contents: 150,000 IU vitamin A, 56,500 IU vitamin D_3_, and 8,000 IU vitamin E; for trace mineral elements, 14 mg selenium (selenite form), 80 mg iodine (iodide form), 290 mg copper sulfate, 1,925 mg manganese sulfate, 2,050 mg zinc oxide, as well as 24 mg cobalt sulfate. Digestible crude protein (CP) values of experimental diets were computed in reference to the agricultural industry standard NY/T 816-2021 based on metabolizable energy (ME), whereas all other nutritional indicators were determined through laboratory testing.

### 2.3 Sample collection and measurement

At the conclusion of the trial, five rams from each group were randomly selected after a 12-h feed fast and a 2-h water fast. The animals were stunned using a captive bolt pistol and subsequently exsanguinated humanely, following the approved animal welfare procedures. Subsequently, 200 g of the longissimus dorsi muscle samples were collected from the 12th-13th ribs. These samples were vacuum-packed, quick-frozen in liquid nitrogen, and then stored at −80°C.

#### 2.3.1 Meat quality

Meat quality was evaluated by determining physical parameters (pH, color, water loss, and cooking loss) and chemical components (moisture, fat, protein, and ash). The pH was recorded using a calibrated portable pH meter (PHBJ-261L). Color attributes (L^*^ for lightness, a^*^ for redness, b^*^ for yellowness) were measured with a color meter (DC-700C-1) under dark conditions. For water loss measurement, a 1-cm-thick sample of the longissimus dorsi muscle was cut vertically to the fiber direction using a 3-cm-diameter circular punch (DL-100). The sample was weighed (G1), then pressed between two layers of filter paper under a constant pressure of 35 kg using a mechanical press (YL-100, Yuelong Testing Instruments Co., Ltd., China) for 5 min at room temperature (25 ± 2 °C), and reweighed immediately after pressing (G2). Water loss rate was derived as (G1–G2)/G1 × 100%. Cooking loss was assayed by taking approximately 100 g of meat, trimming excess fat, weighing (W1), steaming at 100 °C for 45 min, cooling for 30 min, and reweighing (W2). The cooking loss percentage was calculated as (W1–W2)/W1 × 100%.

The contents of moisture, crude protein, crude fat and ash in the meat were determined using AOAC methods (22). Moisture content was determined by completely drying the samples in an oven at 105 °C. Crude protein was determined by the Kjeldahl method, crude fat was determined by Soxhlet extraction, and ash content was calculated as the weight of inorganic material left after samples were burned in a muffle furnace at 550 °C for 4 h.

#### 2.3.2 Fatty acid extraction and measurement by GC/MS

Approximately 5 g of meat sample was mixed with 2 mL of 95% ethanol containing 100 mg of pyrogallic acid, followed by the addition of 10 mL of 1 mol/L hydrochloric acid, and the mixture was thoroughly homogenized. Samples were hydrolyzed at 70–80 °C for 40 min, then cooled and mixed with 10 mL of 95% ethanol and transferred to a separatory funnel. Extracts were rinsed with 50 mL of a mixture of ethyl ether and petroleum ether, and then dried in an oven at 100 ± 5 °C for 2 h. Before methyl esterification, 1.0 mL of internal standard stock solution (5 mg/mL, equivalent to 5 mg of triundecanoin, C11:0 TAG, Sigma-Aldrich) was accurately added to each sample extract and mixed thoroughly. For fatty acid extraction, 2 mL aliquots of 2% sodium hydroxide methanol solution were added followed by 3 mL of 14% boron trifluoride in 95% methanol and 1 mL of n-hexane in a glass centrifuge tube, and the extract was shaken for 30 min at 85 °C. The solution was re-extracted with 1 mL of n-hexane by shaking for 2 min. After the final extraction, a 100-μL aliquot of the supernatant was diluted with n-hexane to 1 mL. The solution was filtered through a 0.45-μm membrane filter and then injected onto a TG-5MS gas chromatograph column (30 m × 0.25 mm × 0.25 μm). The column temperature program was as follows: held at 80 °C for 1 min, then raised to 200 °C at 10 °C/min, subsequently to 250 °C at 5 °C/min, and finally to 270 °C at 2 °C/min, where it was maintained for 3 min. The injector temperature was 290 °C. The carrier gas was high-purity helium (He, purity ≥99.999%) in constant flow mode at a flow rate of 1.2 mL/min, with a splitless injection mode. For mass spectrometry, the ion source temperature was 280 °C, the transfer line temperature was 280 °C, the solvent delay time was 5 min, the scanning range was 30–400 amu, and the EI ion source was set at 70 eV. Qualitative analysis was performed by comparing retention times and mass spectra with a 37-component FAME mixed standard (Supelco 37 Component FAME Mix, Sigma-Aldrich, USA) and the NIST library. Quantitative analysis was conducted using the internal standard method according to the Chinese National Standard GB 5009.168-2016.

#### 2.3.3 Amino acid assay by HPLC

Five-g samples were thawed and mixed with 10 mL of 6 M HCl. To prevent oxidative degradation, the tubes were flushed with high-purity nitrogen for 3 min (repeated three times) and immediately sealed by flame-melting the glass, then hydrolyzed at 110 °C for 22 h. Once cooled, 1 mL of filtrate was dried under reduced pressure at 40 °C, dissolved in 1 mL of sodium citrate buffer (pH 2.2), and filtered through a 0.22 μm membrane. Because tryptophan (Trp) is completely degraded under acidic high-temperature conditions, a separate alkaline hydrolysis using 4 M LiOH (with 5% starch as an antioxidant) at 110 °C for 20 h under the same nitrogen protection was employed for Trp determination, followed by neutralization to pH 7.0. Amino acids were analyzed using an automated amino acid analyzer equipped with a sulfonic acid cation-exchange resin column (135 ± 5 °C). Quantification was based on a mixed amino acid standard (Sigma-Aldrich, AAS18) and an L-Trp standard (Sigma-Aldrich, T0254). The elution gradient used stepwise citrate-sodium citrate buffers (pH 3.3, 4.9, and 6.0) at a flow rate of 0.4 mL/min, with ninhydrin delivered at 0.35 mL/min. A 20 μL volume was injected, and detection was performed at 570 nm (primary amino acids) and 440 nm (proline).

#### 2.3.4 IMP determination in muscle

IMP was extracted from 2-g muscle samples by homogenizing three times in 20 mL of 5% perchloric acid, followed by centrifugation at 799 × g for 10 min at 4 °C (Eppendorf 5430R, Germany). The combined supernatants were neutralized to pH 6.5 with KOH, brought to 50 mL with distilled water, filtered through Whatman No. 1 paper to remove potassium perchlorate precipitate, an d passed through a 0.45 μm membrane. For quantification, an IMP standard (Sigma-Aldrich, USA) was dissolved in ultrapure water and serially diluted to establish a calibration curve. A spiked recovery test was performed prior to extraction, yielding an average recovery of 98.5% (RSD <2%). The filtrate was analyzed by HPLC using a C18 column (250 × 4.6 mm, 5 μm) at 25 °C. The mobile phase consisted of 3.5 mL phosphoric acid, 200 mL water, 7.2 mL triethylamine (pH 6.5), and 50 mL methanol, degassed by ultrasonication, at a flow rate of 1 mL/min. The injection volume was 20 μL, and detection was at 254 nm.

IMP was extracted from 2-g muscle samples by homogenization three times in 20 mL of 5% perchloric acid, and the homogenates were centrifuged at 799 × g for 10 min at 4 °C. The combined supernatants were neutralized to pH 6.5 with KOH, brough to a final volume of 50 mL with distilled water, and then filtered through a 0.45 μm membrane. The filtrate was analyzed by HPLC using a C18 column (250 × 4.6 mm, particle size 5 μm) at 25 °C. The mobile phase consisted of a mixture of 3.5 mL of phosphoric acid, 200 mL of water, 7.2 mL of triethylamine (pH 6.5) and 50 mL of methanol, degassed by ultrasonication and delivered at a flow rate of 1 mL/min. The injection volume was 20 μL, and detection was performed at 254 nm.

#### 2.3.5 Relative expression levels of IMP-related genes

The mRNA sequences of the genes to be tested were retrieved from NCBI and suitable primers were designed, screened, and synthesized by Prime Genetics (Table 3). Muscle tissues from the CON, SBS24 and SBS30 groups were ground using a cryomill (JXFSTPRP-II, Shanghai Jingxin Technology Co., Ltd., Shanghai, China), and RNA was extracted with TRIzol. The RNA concentration and purity were determined by NanoDrop, and the RNA was reverse transcribed into cDNA using a reverse transcription kit (Tiangen Biochemical Technology Beijing Co., Beijing, China). The cDNA templates were subjected to real-time fluorescent quantitative PCR with SYBR Green fluorescent detection reagent on a Bio-Rad CFX96 instrument. Amplification program: initial denaturation at 95 °C for 10 min; 40 cycles consisting of 95 °C denaturation for 15 s and 60 °C annealing extension for 1 min (fluorescence signal collection at extension stage). At the end of the run, the relative expression levels of the selected genes were calculated and normalized to the GAPDH gene as an internal reference, and the Ct values were obtained using the 2^−ΔΔ*Ct*^ method.

**Table 3:** RT-qPCR primer sequences.

GenBank/RefSeq	Gene	Primer sequences (5' → 3')	Product size/bp	Annealing temperature, °C
NC_056056.1	*GAPDH*	F: GGCGTGAACCACGAGAAGTA	141	55.04
		R: GGCGTGGACAGTGGTCATAA		
NC_091727.1	*PRPS1*	F: GCAGCTCCCACCAGGATCTA	110	55.39
		R: TCCGATTTCCACACAGGTCTC		
NC_091247.1	*ADSL*	F: CGGCCTGTGAGTACGATAGC	148	54.82
		R: AAACCCAGTGTCTGCTCGG		
NC_056055.1	*ATIC*	F: ATTTGCCGGAATCTGCGTTC	107	55.16
		R: GATGCCAATGACCTGCCCAT		
NC_056054.1	*AMPD1*	F: TTTCCTCAGGCACATCACGG	101	55.21
		R: TGGTCCACTCCTGGGGTTTA		
NC_030828.1	*ADSSL1*	F: TGACCTTGACTTCGGGACCT	173	55.15
		R: TCTCGTTGATCTGCTCGGTG		
NC_091248.1	*IMPDH1*	F: GACTTCAGCCCTGACTCGAAA	112	55.88
		R: GATACCGCCCATCAGAGCCA		

F, forward primer; R, reverse primer.

### 2.4 Statistical analysis

All raw experimental data were sorted in Excel 2019 software. One-way analysis of variance (ANOVA) was carried out using SPSS 26.0, and Duncan multiple range tests were applied to compare inter-group differences. Linear and quadratic regression models were established to analyze the changing trends of each indicator with gradient SBS supplementation. All experimental results were presented as mean ± standard error of the mean (SEM). *P* < 0.05 represented statistically significant differences between treatments, while *P* < 0.01 indicated extremely significant inter-group discrepancies.

## 3 Results

### 3.1 Meat quality

The effects of increasing dietary SBS levels on the edible quality of the longissimus dorsi muscle of rams are presented in Table 4. Compared with the CON group, the SBS24 group exhibited a significantly higher pH than all other groups (*P* < 0.05). Regarding meat color, the L^*^ value (lightness) was significantly lower in the SBS24 group, while the b^*^ value (yellowness) was significantly lower in both the SBS18 and SBS24 groups relative to the CON group (*P* < 0.05). Furthermore, b^*^ showed a quadratic response, initially decreasing and then increasing with increasing SBS level (*P* = 0.012). In contrast, the a^*^ value (redness) in the SBS18 and SBS24 groups was significantly higher than in the CON, SBS6, and SBS12 groups, and exhibited a linear increase with rising SBS supplementation (*P* = 0.005). Cooking loss was significantly reduced in the SBS18 and SBS24 groups compared with the CON group (*P* < 0.05), and also displayed a linear decreasing trend with increasing SBS level (*P* = 0.043). No significant differences in water loss rate were observed among the other groups (*P* > 0.05).

**Table 4:** Effect of increasing SBS addition on sheep meat quality (*n* = 5).

Items	Groups						SEM	*P*-values		
	CON	SBS6	SBS12	SBS18	SBS24	SBS30		Treat-ment	Linear	Qua-dratic
pH and Colour										
Ph	6.23^b^	6.25^b^	6.24^b^	6.25^b^	6.32^a^	6.24^b^	0.008	0.021	0.078	0.300
Luminosity(L^*^)	56.43^a^	50.99^ab^	47.15^ab^	44.13^ab^	39.80^b^	49.96^ab^	1.803	0.016	0.054	0.053
Redness(a^*^)	12.32^b^	12.67^b^	13.18^b^	16.15^a^	16.59^a^	14.28^ab^	0.468	0.013	0.005	0.132
Yellowness(b^*^)	4.00^a^	3.67^abc^	3.31^abc^	3.15^bc^	2.86^c^	3.79^ab^	0.121	0.047	0.109	0.012
Water holding capacity										
Water loss (%)	30.22	29.09	29.17	28.55	27.80	29.12	0.379	0.632	0.224	0.318
Cooking loss (%)	41.14^a^	40.91^ab^	40.22^abc^	38.61^c^	38.81^bc^	40.17^abc^	0.319	0.034	0.043	0.091

For each row of data, the absence of letters or the presence of identical letters indicates no significant difference (*P* > 0.05), whereas different lowercase and uppercase letters represent significant (*P* < 0.05) and highly significant (*P* < 0.01) differences, respectively.

### 3.2 Muscle composition

The effects of increasing dietary SBS inclusion on the chemical composition of the longissimus dorsi muscle are shown in Table 5. The SBS24 group had a significantly greater crude protein content than the CON, SBS6, SBS12, and SBS30 groups (*P* < 0.05), but not significantly different from that of the SBS18 group (*P* > 0.05). In contrast, there were no significant differences in moisture or crude ash content among any of the treatment groups (*P* > 0.05).

**Table 5:** Effect of increasing SBS addition on sheep longissimus dorsi muscle parameters (DM basis, *n* = 5).

Items	Groups						SEM	*P*-values		
	CON	SBS6	SBS12	SBS18	SBS24	SBS30		Treat-ment	Linear	Qua-dratic
Moisture content (MC)	76.44	75.31	75.91	74.84	74.34	76.27	1.695	0.315	0.443	0.123
Ether extract (EE)	1.99	1.98	1.86	1.87	1.82	1.95	0.060	0.968	0.854	0.727
Crude protein (CP)	19.69^b^	20.48^b^	20.11^b^	21.61^ab^	22.76^a^	20.26^b^	0.294	0.015	0.026	0.059
Crude ash (Ash)	4.68	4.41	4.50	4.56	4.69	4.66	0.090	0.924	0.844	0.950

In a row of data, there is no significant difference between groups without letters or with the same letters (*P* > 0.05), while different lowercase letters indicate significant differences (*P* < 0.05), and different uppercase letters indicate extremely significant differences (*P* < 0.01).

### 3.3 Effects of graded dietary SBS levels on the fatty acid profile of the longissimus dorsi muscle in rams

As shown in Table 6, 15 fatty acids were identified, including eight saturated and seven unsaturated fatty acids. The palmitic acid content in the SBS18 and SBS24 groups was extremely significantly lower compare to CON, SBS6 and SBS30 groups (*P* < 0.01), and the palmitic acid content showed a quadratic curvilinear change of decreasing and then increasing with increasing levels of SBS (*P* = 0.003). The stearic acid content was significantly lower in the SBS24 group than in the CON group (*P* < 0.05). The oleic acid content was significantly higher in the SBS24 group than in the other groups (*P* < 0.05) and was highly significantly greater than in the CON, SBS6, SBS12, and SBS30 groups (*P* < 0.01). The linoleic acid content was significantly higher in the SBS24 group relative to CON (*P* < 0.05), and with increasing SBS, the linoleic acid content showed a gradual linear increase (*P* = 0.048). A significant increase in linolenic acid content was observed in the SBS groups compared with the CON group (*P* < 0.05), and a quadratic relationship with dietary SBS level was detected (*P* = 0.016). The SFA content in the SBS18 and SBS24 groups was highly significantly reduced relative to the CON group (*P* < 0.01), and the SFA levels varied quadratically, decreasing and then increasing as SBS increased (*P* = 0.032). The SBS24 group exhibited highly significantly higher levels of total UFAs, MUFAs, and PUFAs than the CON and SBS30 groups (*P* < 0.01). Both UFA and MUFA contents followed a quadratic curve (initial increase followed by a decrease) with rising SBS inclusion (*P* < 0.01), and the PUFA content also displayed a similar quadratic trend, first increasing and then decreasing (*P* = 0.002 and *P* = 0.006 for the quadratic and linear components, respectively).

**Table 6:** Effect of increasing dietary level of SBS on fatty acid content of longissimus dorsi muscle of rams (*n* = 5).

Fatty acids (% of total)	Groups						SEM	*P*-values		
	CON	SBS6	SBS12	SBS18	SBS24	SBS30		Treat-ment	Linear	Qua-dratic
C10:0	0.19	0.18	0.18	0.17	0.16	0.19	0.004	0.228	0.188	0.125
C12:0	0.15	0.14	0.14	0.13	0.12	0.15	0.017	0.417	0.451	0.131
C14:0	3.03	2.93	2.94	2.84	2.79	2.79	0.036	0.319	0.125	0.779
C14:1	0.09	0.11	0.10	0.12	0.13	0.10	0.006	0.478	0.324	0.212
C15:0	0.39	0.35	0.33	0.33	0.36	0.34	0.022	0.635	0.344	0.197
C16:0	27.85^Aa^	26.63A^Bab^	25.92^BCb^	23.23^Cc^	23.30^Cc^	26.80^ABab^	0.384	<0.001	0.101	0.003
C16:1	2.01	2.04	2.14	2.02	2.17	2.04	0.043	0.886	0.634	0.583
C17:0	1.30	1.18	1.12	1.31	1.28	1.30	0.038	0.667	0.542	0.404
C18:0	21.62^a^	20.91^ab^	21.09^ab^	20.64^ab^	20.03^b^	21.35^ab^	0.199	0.043	0.269	0.121
C18:1n9c	39.83^Bc^	40.29^Bc^	40.90^Bbc^	42.16^ABb^	44.02^Aa^	40.44^Bc^	0.336	<0.001	0.103	0.006
C18:2n6c	2.97^b^	3.01^ab^	3.00^ab^	3.07^ab^	3.65^a^	2.99^ab^	0.080	0.003	0.048	0.306
C20:0	0.12	0.12	0.12	0.11	0.10	0.12	0.006	0.921	0.530	0.575
C20:1	0.05	0.07	0.07	0.09	0.09	0.07	0.005	0.141	0.141	0.085
C18:3n3	0.14^b^	0.20^a^	0.26^a^	0.28^a^	0.32^a^	0.24^a^	0.021	<0.001	0.201	0.016
C20:4n6	0.14	0.17	0.17	0.16	0.17	0.14	0.007	0.693	0.937	0.173
TFA	99.88	98.33	98.49	98.66	98.79	99.06	0.349	0.157	0.506	0.030
ΣSFA	54.65^Aa^	52.44^ABb^	51.85^Bb^	48.75^Cc^	48.14^Cc^	53.03^ABab^	0.486	<0.001	0.085	0.001
ΣUSFA	45.24^Cc^	45.89^BCc^	46.64^BCbc^	47.91^Bb^	50.65^Aa^	46.03^BCc^	0.427	<0.001	0.054	0.002
ΣMUFA	41.98^Bc^	42.51^Bc^	43.22^Bbc^	44.39^ABb^	46.41^Aa^	42.66^Bbc^	0.372	<0.001	0.068	0.006
ΣPUFA	3.26^Bb^	3.38^Bb^	3.42^Bb^	3.51^Bb^	4.24^Aa^	3.37^Bb^	0.077	<0.001	0.103	0.053

Within each row, values with no superscript letters or with the same superscript letters are not significantly different (*P* > 0.05). Different uppercase letters indicate highly significant differences (*P* < 0.01), while different lowercase letters indicate significant differences (*P* < 0.05).

SEM, standard error of the mean; TFAs, total fatty acids; SFAs, total saturated fatty acids; USFAs, total unsaturated fatty acids; MUFAs, total monounsaturated fatty acids; PUFAs, total polyunsaturated fatty acids. ΣTFA = sum of all fatty acids listed in the table.

ΣSFA = C10:0 + C12:0 + C14:0 + C15:0 + C16:0 + C17:0 + C18:0 + C20:0.

ΣUSFA = C14:1 + C16:1 + C18:1n9c + C18:2n6c + C18:3n3 + C20:1 + C20:4n6.

ΣMUFA = C14:1 + C16:1 + C18:1n9c + C20:1.

ΣPUFA = C18:2n6c + C18:3n3 + C20:4n6.

### 3.4 Effects of graded dietary SBS levels on the amino acid profile of the longissimus dorsi muscle in rams

As shown in Table 7, a total of 18 amino acids were determined in this experiment. Compared with the CON group, the serine, valine, ornithine, lysine and tyrosine content were significantly higher in the SBS24 group (*P* < 0.05), while the tryptophan concentration was highly significantly greater (*P* < 0.01). With increasing SBS, there was a gradual and linear increase in serine (*P* = 0.004), a quadratic curve change in tyrosine with a rise and then a fall (*P* = 0.031), and a gradual linear increase in tryptophan with increasing SBS (*P* = 0.035). Compared with the CON group, significantly higher levels of leucine and arginine were observed in the SBS-supplemented groups (*P* < 0.05). The SBS24 group showed a highly significant elevation in both amino acids relative to CON (*P* < 0.01). Furthermore, leucine concentration exhibited a quadratic curvilinear pattern, increasing at first and then decreasing with rising SBS inclusion (*P* = 0.029). Arginine showed a gradual linear increase with % SBS (*P* = 0.011). The contents of total amino acids (TAA), essential amino acids (EAA), and non-essential amino acids (NEAA) in the SBS24 group were significantly higher than those in the CON group (*P* < 0.05). In addition, TAA content exhibited a linear increase with increasing SBS supplementation (*P* = 0.037).The content of amino acids associated with meat freshness and flavor appeared in descending order: SBS24>SBS18>SBS12>SBS6>SBS30>CON (*P* > 0.05), and the fresh-flavor amino acid content changed according to a quadratic curve with increasing SBS (*P* = 0.044).

**Table 7:** Effects of graded dietary SBS levels on the amino acid profile of the longissimus dorsi muscle in rams (*n* = 5).

Amino acid (mg/100g)	Group						SEM	*P*-value		
	CON	SBS6	SBS12	SBS18	SBS24	SBS30		Treat-ment	Linear	Quad-ratic
Gly	216.40	216.92	217.83	218.13	227.20	216.04	4.440	0.561	0.769	0.814
Ala	550.20	557.33	558.87	580.47	600.59	551.79	12.982	0.361	0.578	0.513
Ser	75.73^b^	66.72^b^	69.32^b^	71.12^b^	97.36^a^	75.63^b^	2.810	0.003	0.004	0.715
Pro	40.69	43.95	41.89	42.40	53.73	40.84	1.772	0.063	0.393	0.486
Val	34.95^b^	34.96^b^	37.43^ab^	36.24^b^	42.27^a^	37.27^ab^	0.773	0.040	0.030	0.488
Thr	55.28	56.25	57.89	61.60	63.93	56.17	1.448	0.142	0.305	0.222
Ile	29.03	29.63	29.88	31.22	33.88	30.39	0.749	0.114	0.190	0.530
Leu	46.68^Bb^	57.66^ABa^	57.66^ABa^	58.27^ABa^	65.41^Aa^	57.92^ABa^	1.813	0.009	0.104	0.029
Asn	50.76	51.78	51.23	53.30	59.59	55.92	2.052	0.310	0.259	0.937
Orn	10.66^b^	11.26^b^	11.23^b^	13.03^ab^	20.10^a^	11.80^b^	1.155	0.045	0.139	0.515
Lys	61.27^b^	61.94^b^	62.30^b^	62.41^b^	85.66^a^	61.44^b^	3.032	0.013	0.220	0.605
Glu	26.39	27.41	27.92	29.90	33.74	28.01	1.159	0.123	0.239	0.446
Met	22.57	23.69	24.51	24.76	25.77	23.04	0.631	0.227	0.517	0.222
His	233.48	238.82	252.11	253.85	269.05	242.65	9.844	0.400	0.529	0.530
Phe	39.30	39.61	41.08	41.49	43.06	39.44	0.697	0.192	0.440	0.242
Arg	76.22^Bb^	94.96^ABa^	95.91^ABa^	98.85^ABa^	102.32^Aa^	97.65^ABa^	2.637	0.007	0.011	0.052
Tyr	36.85^b^	39.84^ab^	41.67^ab^	43.33^ab^	44.69^a^	38.00^ab^	1.009	0.018	0.282	0.031
Trp	7.33^Bb^	7.61^Bb^	8.17^ABb^	7.88^ABb^	10.61^Aa^	8.04^ABb^	0.315	0.022	0.035	0.362
TAA	1608.99^Bb^	1660.35^ABb^	1686.92^ABb^	1728.25^ABab^	1878.96^Aa^	1672.05^ABb^	25.746	0.005	0.037	0.126
EAA	296.40^Bb^	311.34^ABb^	318.92^ABab^	323.89^ABb^	370.59^Aa^	313.72^ABb^	7.063	0.004	0045	0.160
NEAA	1286.04^b^	1320.49^b^	1324.91^ab^	1339.91^ab^	1370.55^a^	1480.60^b^	21.031	0.017	0.095	0.192
DAA	956.82	988.26	993.45	1023.97	1068.13	987.42	15.201	0.071	0.184	0.044

Within each row, values without a common superscript differ: uppercase letters denote *P* < 0.01; lowercase denote *P* < 0.05; same/no letter means *P* > 0.05. SEM: standard error of the mean. Abbreviations: Gly (glycine), Ala (alanine), Ser (serine), Pro (proline), Val (valine), Thr (threonine), Ile (isoleucine), Leu (leucine), Asn (asparagine), Orn (ornithine), Lys (lysine), Glu (glutamic acid), Met (methionine), His (histidine), Phe (phenylalanine), Arg (arginine), Tyr (tyrosine), Trp (tryptophan). TAA (total amino acids), EAA (essential), NEAA (non-essential), DAA (flavor-enhancing). ΣTAA = total of all AAs; ΣEAA = Val+Leu+Ile+Phe+Lys+Met+Thr+Trp; ΣNEAA = TAA – ΣEAA; ΣDAA = Glu+Ala+Arg+Tyr+Asn+Gly.

### 3.5 Effects of increasing dietary addition of SBS on the IMP content of the longissimus dorsi muscle of rams

The IMP content in the SBS24 group was significantly higher than that in the CON and SBS30 groups (*P* < 0.05), whereas no significant differences were observed among the other groups (*P* > 0.05; Figure 1).

**Figure 1:**
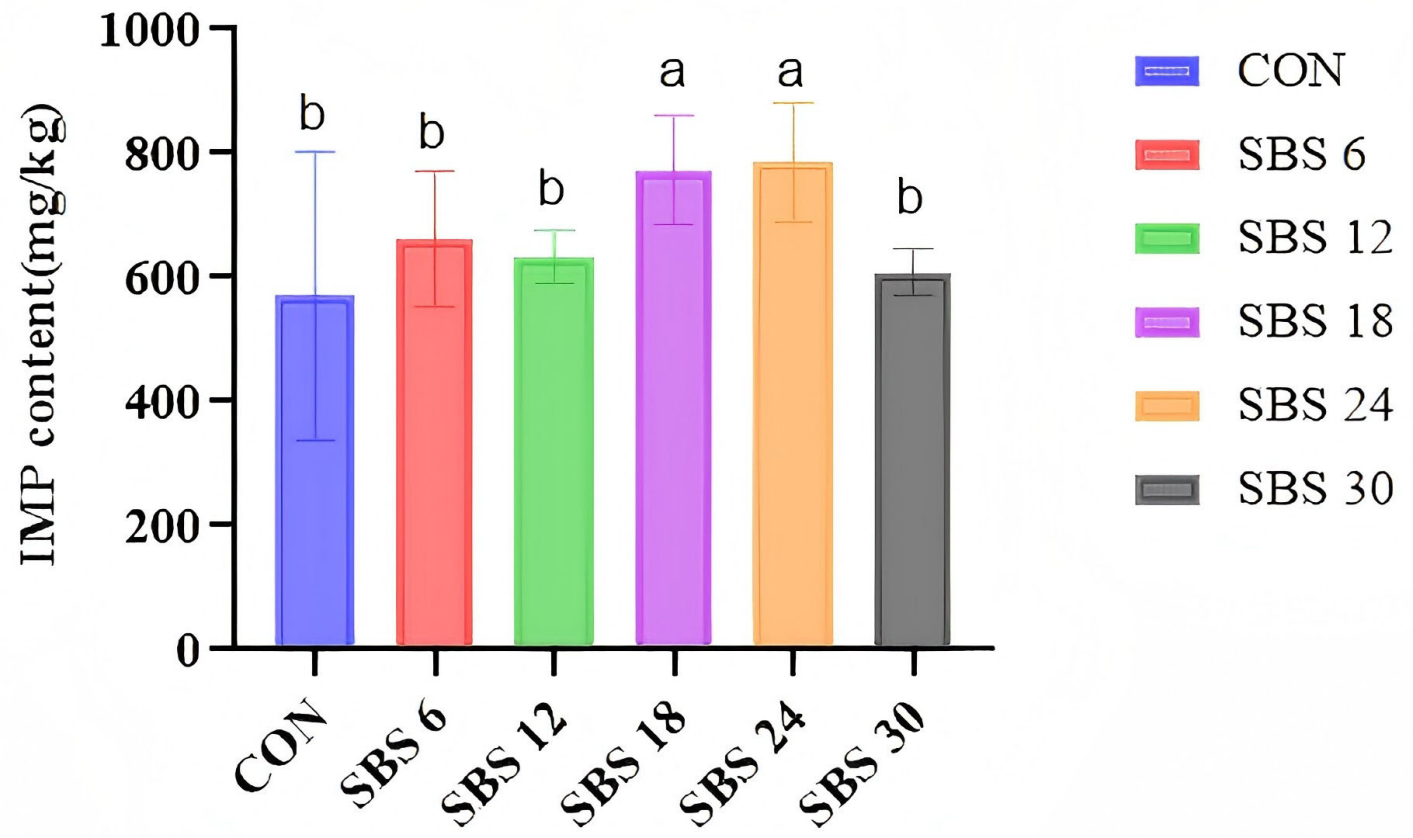
Effect of increasing dietary SBS addition on IMP content of longissimus dorsi muscle of rams.

### 3.6 Effects of increasing SBS levels on the relative expression of IMP-related genes in the longissimus dorsi muscle of rams

Figure 2 shows that the relative expression levels of PRPS1, ADSL, ATIC, and AMPD1 were highly significantly increased in the SBS24 group compared with the CON and SBS30 groups (*P* < 0.01), and a highly significant elevation of PRPS1 was also observed in the SBS30 group relative to CON (*P* < 0.01). In contrast, ADSSL1 expression was highly significantly decreased in both SBS24 and SBS30 groups vs. CON (*P* < 0.01). Moreover, IMPDH1 expression in the SBS24 group was highly significantly lower than in CON (*P* < 0.01) and significantly lower than in SBS30 (*P* < 0.05).

**Figure 2:**
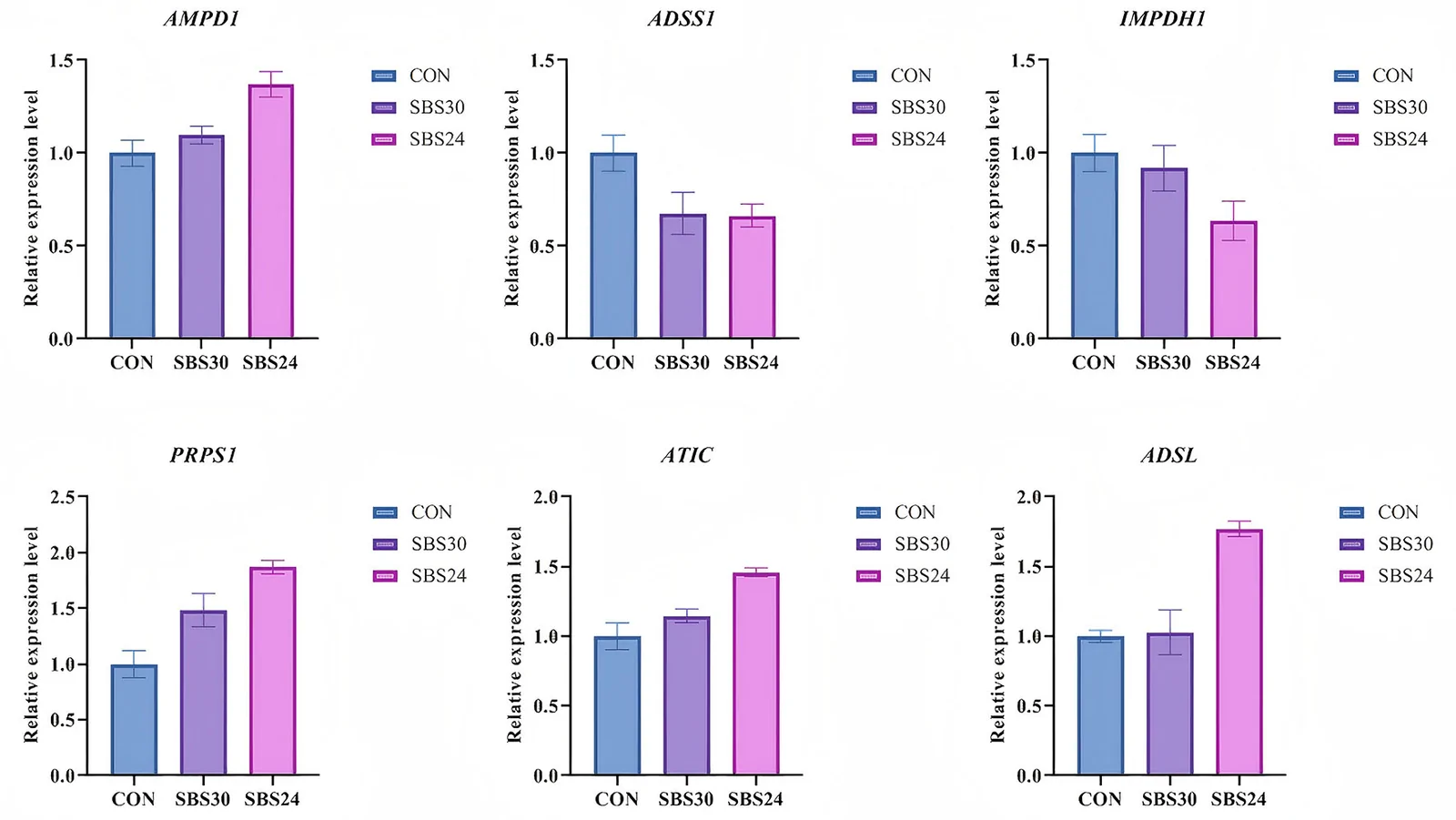
Effect of increasing SBS content on the relative expression level of IMP-related genes in the longissimus dorsi muscle of rams.

## 4 Discussion

### 4.1 Effects of SBS on meat quality of Bainuburke rams

The pH of muscle reflects the rate and intensity of glycolysis following slaughter and directly affects its water-holding capacity and meat color (23). A higher pH slows down glycolysis in muscle glycogen, reduces water loss, enhances protein stability, and helps the meat retain its fresh, succulent flavor for a longer period (24). Earlier studies showed that adding flavonoid-rich sand onion to the diet of small-tailed cold sheep significantly raised the pH at 24 h, which in turn reduced cooking water loss—consistent with the principle that higher pH helps meat retain moisture and freshness (25). The results of this study showed that the pH value of the SBS24 group was significantly higher than that of the CON group, while cooking loss was significantly lower than that of the CON group, indicating that appropriate addition of SBS can slow down the spoilage rate of Bainuburke mutton, extend its shelf life, and improve its flavor. This may be because SBS is rich in antioxidants such as flavonoids, which can improve meat quality by enhancing cell membrane stability, reducing calcium levels, raising pH, promoting the transport of Ca^2^? into muscle cells (26), slowing the rate of glycolysis, and preventing the oxidative denaturation of myofibrillar proteins (27). However, adding more than 24% SBS may have the opposite effect.

Meat color is a visual manifestation of the physiological and biological characteristics of muscle tissue, and it plays a crucial role in consumer acceptance and purchasing decisions. A good color and sheen can stimulate consumers' desire to purchase. Meat color is influenced by myoglobin and hemoglobin, and is susceptible to the negative effects of oxidation. Myoglobin can be converted to oxygenated myoglobin, and its ratio can directly affect the a^*^ (redness) value (28). Better meat quality is a function of higher a^*^ values, whereas higher L^*^ values and lighter color are indicative of poorer meat quality. The b^*^ values are affected by dietary intake of antioxidants like carotenoids in the feed as well as the content of intermuscular and intramuscular fat (29). A previous study showed that the addition of 800 mg/kg apple polyphenols to pig rations reduced L^*^ and b^*^ values in the longissimus dorsi muscles of fattening pigs (30). In this study, compared to the CON group, the SBS24 group had significantly reduced brightness and yellowness; conversely, the rødness of both the SBS18 and SBS24 groups was significantly increased. These results align with those reported by Hugh et al. (31), where the addition of wheat straw to the diet of Tibetan sheep increases the a^*^ value and reduces the L^*^ and b^*^ values. This suggests that, unlike conventional plant polyphenols (such as apple polyphenols), the characteristic flavonoids in SBS possess distinct structural and functional specificity (32): their phenolic hydroxyl groups or glycosidic substituents at the C-7 position can not only chelate metal ions to inhibit free radical generation, but baicalein also exerts potent antioxidant effects via the Nrf2/HO-1 pathway (33), while baicalin alleviates oxidative stress via the NF-κB and Nrf2-HO1 pathways (34). These combined effects reduced the oxidative damage to myoglobin caused by lipid peroxides, enhanced myoglobin reductase activity, and delayed the oxidation of myoglobin (35).

It is noteworthy that the meat color parameters of the SBS30 group were inferior to those of the SBS24 group. This adverse effect induced by high-dose SBS (>24%) can be attributed to three physiological mechanisms: first, the “pro-oxidant” effect of excessive flavonoids disrupted the redox balance and induced the production of reactive oxygen species (ROS) (36); second, excessive inhibition of rumen microorganisms disrupted the microecological balance and reduced fermentation function (37); and third, excessive intake of flavonoids increased the metabolic and detoxification burden on the liver (38). These factors ultimately undermined the antioxidant benefits, confirming that the dietary supplementation ratio must be strictly optimized when using SBS as an unconventional forage for sheep.

### 4.2 Effects of SBS on fatty acids and amino acids in rams

The content and composition of fatty acids in meat have a decisive role in determining meat quality and flavor (39). Unsaturated fatty acids are classified into monounsaturated and polyunsaturated fatty acids, with the latter serving as the primary source of muscle flavor (40). The rich flavor of lamb is a key factor influencing consumer purchasing decisions; its distinctive “gamey” odor primarily results from volatile compounds generated during the oxidation of saturated fatty acids (41). Studies have shown that stearic acid (C18:0) is the main contributor to the strong odor of lamb (42). As an important saturated fatty acid in lamb, changes in C18:0 concentration, along with variations in C16:0, affect the total saturated fatty acid (SFA) content in intramuscular fat (43). Excessive intake of saturated fatty acids can increase blood levels of high-density lipoprotein (HDL) and low-density lipoprotein (LDL), leading to diseases such as coronary atherosclerosis (44). This study found that dietary supplementation with SBS—particularly in the SBS24 group—reduces the content of C16:0 and C18:0 in lamb meat, thereby improving its gamey odor and overall flavor.

Increasing the USFA content could improve the flavor, texture and nutritional value of meat. PUFAs have higher nutritional value and can reduce the level of cholesterol in human blood, thus lowering the risk of cardiovascular diseases (45). The characteristic gamey odor of lamb mainly originates from branched-chain fatty acids (BCFA), particularly 4-methyloctanoic acid and 4-methylnonanoic acid. In this trial, the significant reduction in straight-chain saturated fatty acids including C16:0 and C18:0 in the SBS24 group may have a positive effect on reducing the gamey odor and improving the flavor of lamb. Meanwhile, the increase in unsaturated fatty acids such as C18:1n9c (oleic acid) and C18:2n6c (linoleic acid) not only serves as important flavor precursors (which can generate volatile flavor compounds through oxidation during heating) but also improves muscle tenderness and juiciness.

The reduction of saturated fatty acid content in mutton is closely related to the mechanism of ruminal biohydrogenation. Dietary unsaturated fatty acids are typically extensively hydrogenated into saturated fatty acids by rumen microorganisms (such as Butyrivibrio fibrisolvens). However, the characteristic flavonoids in *Scutellaria baicalensis* stems and leaves (baicalin and baicalein) can selectively inhibit the reductase activity of these ruminal biohydrogenating bacteria, thereby blocking the terminal reaction of biohydrogenation (the conversion of trans-11 C18:1 to C18:0). This intermediate process directly reduces the production of stearic acid (C18:0) in the rumen, subsequently decreasing the absorption and intramuscular deposition of saturated fatty acids (46). Furthermore, at the level of lipid metabolism regulation, baicalin and baicalein can directly target the AMPKα and SREBP-1c signaling pathways: activation of the AMPKα pathway induces the phosphorylation and inactivation of acetyl-CoA carboxylase (ACC), thereby promoting fatty acid β-oxidation and inhibiting *de novo* fatty acid synthesis (47); meanwhile, downregulation of SREBP-1c expression further inhibits the expression of lipogenesis-related genes (48). These specific mechanisms elucidate the mechanisms by which the bioactive substances in *Scutellaria baicalensis* stems and leaves improve fatty acid composition, meat quality, and growth performance.

The type and content of amino acids (AAs) in muscle is an important indicator of its nutritional value, as several amino acids are essential for normal human metabolism. For example, lysine is one of the limiting AAs that must be supplied by the food we eat. It participates in the function of promoting growth, development and maintaining health. In cooking, it can generate thiophene that enhances the flavor of lamb (49). Arginine is another EAA that affects the flavor of lamb, but also helps to maintain energy balance and regulate fatty acid metabolism in humans, thus, both improving meat quality and inhibiting obesity (50). Leucine enhances the body's antioxidant capacity by increasing the activity of antioxidant enzymes (51). Serine is involved in the synthesis of methionine, glycine and cysteine, and plays a role mainly in lipid metabolism, immune surveillance, and operation of the central nervous system.

In this study, the contents of arginine and leucine were significantly higher in the SBS group, suggesting that SBS supplementation can improve the flavor of lamb and promote the health of the body. Based on the significant elevation of muscle crude protein content in the SBS24 group (as detailed in Section 3.2), a clear correlation chain from ruminal-intestinal protein supply to intramuscular amino acid deposition can be established. In ruminants, rumen microbial protein (MCP) serves as the primary source of absorbable amino acids in the small intestine. The characteristic flavonoids in SBS (baicalin and baicalein) reshape the rumen microbial community to enhance MCP synthesis (52), thereby providing an expanded pool of essential amino acids (EAAs) to the small intestine. Upon intestinal absorption, the deposition of EAAs (e.g., leucine and arginine) is primarily governed by the activation of the mTOR signaling pathway, which promotes myofibrillar protein translation and muscle hypertrophy (53). In contrast, the accumulation of flavor-enhancing amino acids (DAAs) involves multi-level regulatory mechanisms: while they increase synchronously with total muscle protein, their free amino acid pool is specifically regulated by amino acid transporters (e.g., SLC1A1 and SLC38A2) to directly contribute to savory taste and serve as Maillard reaction precursors (54). Recent multi-omics studies have confirmed that this “diet-microbiome-muscle axis” directly mediates intramuscular amino acid deposition in ruminants, which elucidates how 24% SBS supplementation optimizes the muscle amino acid profile and significantly elevates crude protein content.

### 4.3 Effects of SBS on the expression levels of inosinic acid and related genes in the longissimus dorsi muscle of rams

After slaughter, the hypoxic environment in the muscle promotes the breakdown of ATP to produce inosine monophosphate (IMP), a key flavor-enhancing substance closely associated with the umami taste of cooked lamb. Furthermore, the degradation products of IMP can undergo Maillard reactions with certain amino acids upon heating, generating volatile aroma compounds that contribute to a meaty flavor. In this study, the IMP content in the SBS24 group was significantly higher than that in the CON and SBS30 groups, indicating that a higher level of SBS effectively promotes IMP synthesis. The observed improvement may be attributed to the characteristic flavonoids in SBS, primarily baicalin and baicalein, which aligns with the nutritional parameters of the SBS raw material measured in this trial. This is consistent with comparative studies indicating that feeding livestock with polyphenol-rich forages can regulate muscle IMP metabolism (55). To attain the best results, however, the dosage of SBS must be carefully calibrated based on the actual production parameters.

According to related studies (56, 57), a specific array of genes (PRPS1, ADSL, ATIC, and AMPD1), plays an important role in IMP synthesis, while ADSSL1 and IMPDH1 genes function in IMP catabolism. Studies have shown that the addition of polyphenol-rich botanicals, such as jasmine residue, to goat rations upregulated the expression of ADSL and ATIC and increased IMP content in meat (58). In this study, the IMP content in the SBS24 group was significantly higher compare to CON and SBS30 groups, and the relative expression levels of PRPS1, ADSL, ATIC, and AMPD1 were highly significant greater than those in the CON and SBS30 groups. Compared with the CON group, ADSSL1 expression was highly significantly decreased, and IMPDH1 expression was significantly decreased relative to both CON and SBS30 groups. The 24% SBS supplementation was associated with higher mRNA levels of PRPS1, ADSL, ATIC, and AMPD1, and lower levels of ADSSL1 and IMPDH1 compared to controls.

Mechanistically, the characteristic flavonoids in SBS (baicalin and baicalein) exert potent antioxidant effects by scavenging reactive oxygen species (ROS) and alleviating oxidative stress in muscle tissue (59). This redox balance subsequently activates the AMP-activated protein kinase (AMPK) signaling pathway, which acts as a central metabolic sensor initiating transcriptional regulation of purine metabolism genes to maintain cellular energy homeostasis (60). Through this AMPK-mediated pathway, SBS flavonoids drive a dual synergistic mechanism governing IMP accumulation. First, the upregulation of *de novo* purine synthesis genes (PRPS1, ADSL, ATIC, and AMPD1) accelerates the enzymatic conversion of ribose-5-phosphate into inosinic acid, actively promoting IMP generation (61). Concurrently, the downregulation of IMP catabolic genes (ADSSL1 and IMPDH1) restricts the metabolic flux of IMP toward AMP or GMP synthesis, effectively reducing IMP degradation (62). This bidirectional regulation—accelerating synthesis while blocking catabolism—maximizes the net deposition of IMP.

Importantly, the improvement of meat flavor relies on the synergistic effects of multiple indicators. In this trial, the 24% SBS supplementation simultaneously optimized meat color, elevated umami amino acid contents (e.g., Asp, Glu, and Gly), and increased IMP deposition. This tripartite synergy comprehensively enhances the umami taste of lamb: a superior meat color reflects favorable muscle pH and antioxidant status, which preserves the structural integrity of flavor precursors; concurrently, the elevated IMP and free umami amino acids directly stimulate taste receptors and interact during cooking to generate rich volatile flavor compounds. Nevertheless, overdosing may reverse this beneficial effect. In summary, a 24% SBS feed supplement was the most effective in up-regulating the expression of IMP synthesis-related genes and down-regulating IMP catabolism-related genes, and resulted in the greatest synergistic improvement in the comprehensive flavor of lamb and mutton.

### 4.4 Research limitations and prospects

Although this study demonstrates the positive effects of dietary SBS supplementation on growth performance, meat quality, and flavor in sheep, several limitations must be acknowledged. First, the experimental period lasted for 80 days (including a 10-day adaptation period), representing a short-term feeding trial. It could not fully assess the cumulative effects of long-term SBS feeding on meat quality and the sustained impact on animal production performance. Future studies are recommended to conduct long-term feeding trials covering the entire fattening cycle (e.g., 120 days or longer) to clarify the optimal SBS supplementation strategy and its temporal mechanisms. Second, this study primarily focused on the expression regulation of key genes in IMP metabolism but lacked direct evidence regarding how the active flavonoids in SBS (such as baicalin and baicalein) enter the bloodstream, target muscle tissues, and regulate the molecular signaling networks of the IMP pathway. Subsequent research could employ multi-omics technologies (transcriptomics, proteomics, and metabolomics) combined with cellular and animal models to deeply elucidate the molecular mechanisms by which active ingredients regulate IMP metabolism and explore their synergistic effects with other flavor precursor substances (such as free amino acids and small peptides).

In conclusion, the 24% SBS supplementation level demonstrated significant potential for improving the nutritional value and flavor of mutton in short-term trials, but its long-term effects and deep molecular mechanisms require systematic study. Expanding research in these directions will help promote the scientific and industrial application of *Scutellaria baicalensis* straw as a functional roughage in the production of high-quality mutton.

## 5 Conclusions

In this study, the inclusion of 24% SBS in the diet was found to enhance lamb and mutton quality, boost IMP deposition, and elevate the levels of protein, fat, essential amino acids, flavor amino acids, C18:1n9c MUFAs, and C18:3n3 PUFAs, collectively improving the nutritional profile of mutton. Based on these findings, a dietary SBS inclusion of 24% is recommended. While further research is needed to fully explore the practical and economic benefits, this SBS addition shows promise for improving animal productivity and human nutrition. It could also potentially contribute to economic gains in the livestock sector and offer environmental advantages through sustainable feed utilization.

## Data Availability

The original contributions presented in the study are included in the article/Supplementary material, further inquiries can be directed to the corresponding author.
