# Supplementary material for: Scutellaria baicalensis straw improves nutritional and flavor quality of mutton via regulating the expression of IMP-related genes
Source: Front Vet Sci. 2026 Sep 23;13:1928789. doi: 10.3389/fvets.2026.1928789 (PMC13643860; doi:10.3389/fvets.2026.1928789)
Supplement: Supplementary file 1 [file Data_Sheet_1.pdf]

Effects of different levels of Scutellaria baicalensis straw on meat quality and expression of IMP-related genes in rams

Supplementary Materials

Figure S1 shows the categories of bioactive substances in SBS. A total of 1,921 bioactive compounds were detected and categorized into 18 types. Among them, flavones accounted for the highest proportion (16.03%), followed by terpenes (14.47%), alkaloids (11.40%), sugars and alcohols (7.65%), amino acids (7.44%), and organic acids (7.24%).

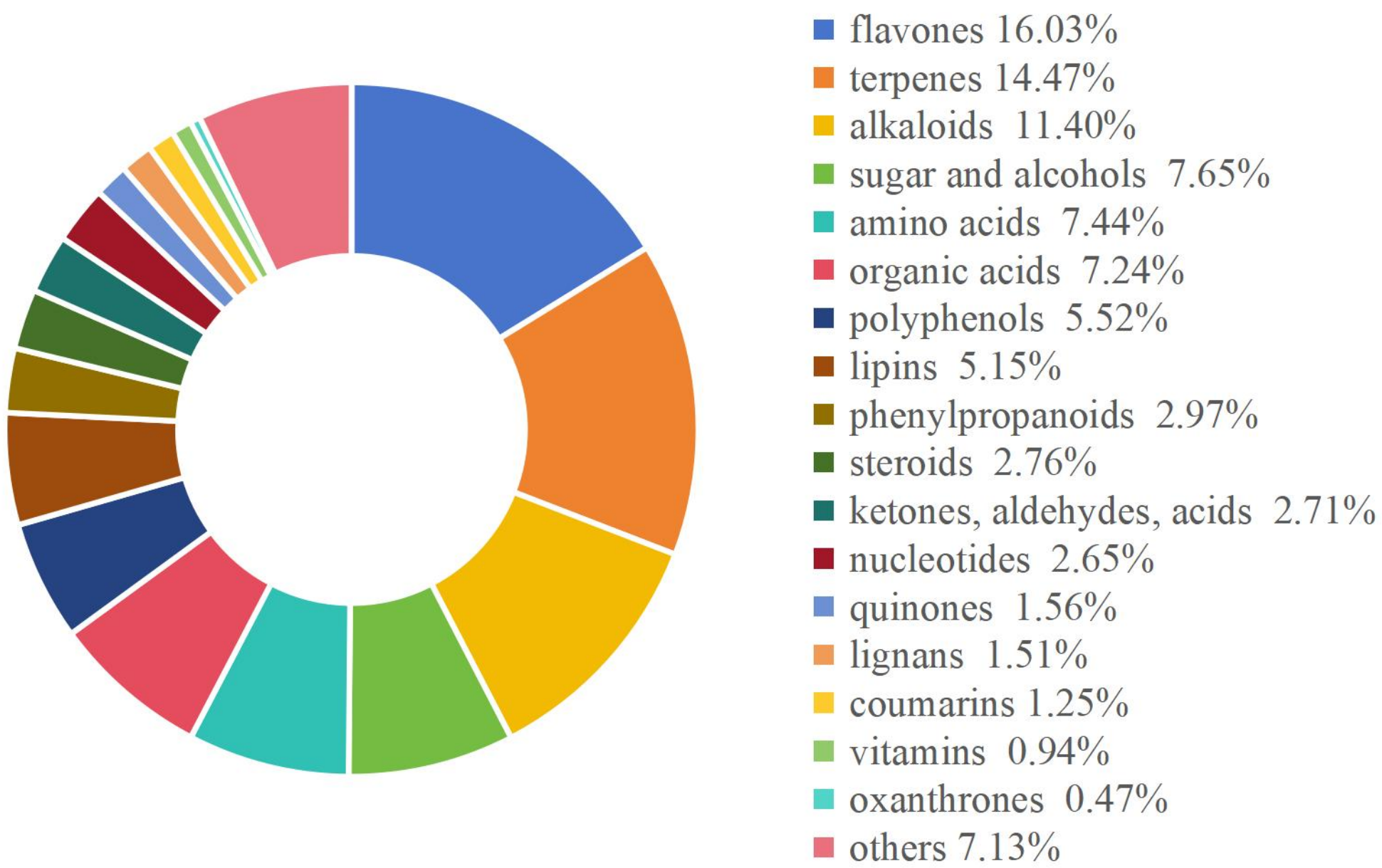

Figure S1. Classification of bioactive substances in SBS
